# They Don’t Want Our Meat-products

**Published:** 1898-01

**Authors:** 


### EDITORIAL.

### THEY DON'T WANT OUR MEAT-PRODUCTS.

THE German Government's report for the six months ending July, 1897, will indicate an increased amount of trichinosis following the eating of American pork. Information seems to point strongly to the inaccuracy of this report, and that the pork raised in Germany and sold under the name of American pork is a much greater factor. This must be the true status of the situation, for the following reasons, well-known to every American veterinarian. First, that only a small percentage of trichinous pork is found under the most careful microscopical investigation by our Government experts. Secondly, the greater proportion of pork consumed in America is not inspected at all, and but very few cases of trichinosis are noted in any part of our country. Statistics of Boards of Health show it to be very rare, indeed. We note also the public clamor of the butchers of Berlin that they must have more protection from the American meat-products, because they cannot compete with them in price.

